# miR-582 negatively regulates pre-B cell proliferation and survival through targeting Hif1α and Rictor

**DOI:** 10.1038/s41419-022-04560-y

**Published:** 2022-02-03

**Authors:** Xinxin Li, Yufei Zhang, Minhua Zheng, Xiuli Cao, Min Guo, Xiangyu Gao, Hua Han

**Affiliations:** 1 Xi’an Key Laboratory of Stem Cell and Regenerative Medicine, Institute of Medical Research, Northwestern Polytechnical University, 710072 Xi’an, Shaanxi P. R. China; 2 Research & Development Institute of Northwestern Polytechnical University in Shenzhen, 518000 Shenzhen, Guangdong P. R. China; 3 State Key Laboratory of Cancer Biology, Department of Biochemistry and Molecular Biology, Fourth Military Medical University, 710032 Xi’an, Shaanxi P. R. China

**Keywords:** Developmental biology, Cell proliferation

## Abstract

B cell development in bone marrow (BM) is a multi-staged process involving pro-B, pre-B, immature B, and mature B cells, among which pre-B cells undergo vigorous proliferation, differentiation, apoptosis, and gene rearrangement. While several signaling pathways participate in pre-B cell development have been clarified, detailed intrinsic mechanisms regulating pre-B cell proliferation and survival have not been fully understood. In the current study, we report that miR-582 regulates pre-B cell proliferation and survival. miR-582 is enriched in pre-B cells. Deletion of miR-582 in mice expanded the BM pre-B cell population in a cell-autonomous manner as shown by competitive BM transplantation. We show that forced miR-582 overexpression inhibited pre-B cell proliferation and survival, whereas downregulation of miR-582 by siRNA significantly promoted pre-B cell proliferation and survival in vitro. We identified that Hif1α and Rictor are authentic targets of miR-582 in pre-B cells as shown by reporter assays. Moreover, miR-582 overexpression reduced the expression of Hif1α and its downstream molecule Glut1, as well as Rictor and mTORC2 activity as shown by attenuated AKT and FoxO1 phosphorylation, while miR-582 knockdown showed opposite effects. miR-582 knockdown-induced increases in pre-B proliferation and survival was abrogated by Hif1α and Rictor inhibitors. Together, miR-582 functions as a negative regulator of pre-B cell proliferation and survival by simultaneously targeting Hif1α and mTORC2 signaling that regulates metabolism in early B cell development.

## Introduction

B lymphocytes (B cells) are pivotal adaptive immune cells that mediate the humoral arm of immune responses against pathogens and mutant cells by secreting antibodies that specifically recognize and bind antigens [1]. In adult mammals, B cells are derived from hematopoietic stem cells (HSCs) accommodated in the bone marrow (BM) [2]. HSCs differentiate into common lymphoid progenitors (CLPs), which select a B cell fate by initiating the consecutive process of DNA recombination of B cell receptor (BCR) genes to generate a specific BCR for each B cell clone [3, 4]. This process is accompanied by staged B cell development including pro-B, pre-B, immature and mature B cells, during which cell proliferation, differentiation, as well as clonal selection take place to guarantee the formation of a proper peripheral B cell repertoire [5]. The pre-B cells, which are divided into large pre-B and small pre-B cells, are characterized by vigorous cell proliferation to provide enough number of B cells for later development [6]. Elucidating mechanisms regulating pre-B cell proliferation and survival is one of the research focuses for understanding B cell development and related human malignancies such as certain types of B cell lymphoma.

B cell development is controlled by both BM microenvironmental cues and cell-intrinsic programs [7]. At the pre-B cell stage, signaling through pre-BCR composed of a rearranged heavy chain and a surrogate light chain is critical for proliferation and survival [8]. Interleukin (IL) 7 derived from BM stromal cells also stimulates pre-B cell proliferation via the IL7 receptor [6, 9]. On the other hand, cell-intrinsic programs such as metabolism also play critical roles in regulating pre-B cell development [10, 11]. For instance, glucose is critically involved in vigorous expansion of pre-B cells [12]. Pre-B cells uptake glucose using the glucose transporter (Glut) 1, and utilize glucose by glycolysis for energy production and biosynthesis [10]. Hypoxia-inducible factor 1 α (Hif1α), a major transcription factor regulating glucose metabolism, is involved in regulating B cell development [13] and T cell activation [14]. Glycolysis in Hif1α deficient BM B cells is attenuated compared with the wild-type control, and the expression of Glut1 and the glycolytic enzyme 6-phosphofructo-2-kinase is greatly reduced [13]. Rictor, the essential component of mammalian target of rapamycin complex 2 (mTORC2), has been reported to regulate B cell maturation [15], although controversy exists on this conclusion [16]. More studies are required to elucidate the exact mechanisms regulating Hif1α/Glut1 and Rictor-mTORC2 signals in B cell development.

MicroRNAs (miRNAs) are short noncoding RNAs of about 22 nucleotides in length that regulate gene expression by binding to the untranslated region (UTR) of mRNAs with seed sequence [17]. Evidence has indicated that miRNAs are involved in B cell development and functions [18]. In an attempt to identify miRNAs involved in organogenesis, we detected miR-582 as a differentially expressed miRNA in proliferating and non-proliferating cells. miR-582 is harbored in the 3rd intron of the mouse phosphodiesterase 4D gene. Previous reports have shown that the expression of miR-582 in B cells of MLL rearranged leukemia patients is significantly lower than other in ALL patients [19]. Compared with normal human B cells, the expression of miR-582 is significantly downregulated in B cells from multiple sclerosis patients [20]. In addition, compared with normal human mature B cells, the expression of miR-582 is dysregulated in B cell chronic lymphocytic leukemia patients [21]. In this study, we show that depletion of miR-582 significantly expands the B cell population in BM by facilitating the proliferation and survival of pre-B cells. Moreover, we demonstrate that miR-582 directly regulates the expression of Hif1α and Rictor to regulate B cell development.

## Materials and methods

### Mice

Four-to-eight-weeks female miR-582 knockout (KO) mice were generated using the CRISPR/Cas9 method by a commercial service provided by Beijing Biocytogen Co. Ltd (Beijing, China). Mice were maintained on the C57BL/6 background in a specific pathogen-free facility, and genotype by polymerase chain reaction (PCR) with tail DNA as a template. Primers are listed in Supplementary Table S1. All animal experiments were approved by the Northwestern Polytechnical University Animal Studies Committee and the Animal Experiment Administration Committee of Fourth Military Medical University, and carried out according to the approved protocols.

### Bone marrow (BM) transplantation

For competitive BM transplantation, 2 × 10^6^ BM cells from WT or miR-582 KO mice (CD45.2) were mixed with equal number of BM cells from normal SJL (CD45.1, Charles River, Beijing, China) mice, and injected into lethally irradiated (9 Gy) recipient mice through the tail vein. Mice were analyzed by flow cytometry 2 months after the transplantation for the proportion of CD45.2^+^ and CD45.1^+^cells [22].

### Fluorescence-activated cell sorting (FACS) assay

Single cell suspensions were prepared from mouse BM, spleen, and lymph nodes by mechanic dispersion, and subjected to erythrolysis according to routine protocols. Cells (3 × 10^5^) in FACS solution (PBS with 2% fetal bovine serum [FBS] and 0.1% NaN_3_) were stained with labeled antibodies in dark for 30 min on ice. After washing, cells were analysis using a FACSCanto II^TM^ flow cytometer (BD Biosciences, San Jose, CA, USA). Cell viability was evaluated with 7-amino-actinomycin D (7-AAD) (BD Biosciences). For cytoplasmic staining, 1 mg/mL GolgiPlug (BD Biosciences) was added 5 h before cell harvest. Cells were incubated with antibodies against surface markers as above, washed, and re-suspended in Cytofix/Cytoperm solution (BD Biosciences) and incubated at 40 °C for 20 min, followed by staining with PE anti-mouse Rictor or PE anti-mouse Hif1α before analysis by flow cytometry. Data were analyzed with the FlowJo7.6 software (Tree Star, USA).

Total B cells were enriched from mouse BM and spleen by using a MACSxpress B cell isolation kit (Miltenyi Biotec, Bergisch Gladbach, Germany). The purity of the enriched B cells was > 90% as determined by FACS after B220 staining. Mouse pre-B cells were further sorted by using a FACSAria II^TM^ cell sorter (BD Biosciences) by gating on CD45^+^IgM^-^B220^+^CD43^−^ cells [23, 24]. The purity of the sorted pre-B cells was > 99% as determined by FACS (Supplementary Fig. S1A).

### Cell culture and transfection

HEK293T cells (American Type Culture Collection, ATCC, Manassas, VA, USA) were maintained in Dulbecco’s modified Eagle’s medium (DMEM) (Invitrogen, Carlsbad, CA, USA) supplemented with 10% FBS, 2 mM L-glutamine, 100 U/mL penicillin and 100 μg/mL streptomycin, and incubated at 37 °C in 5% CO_2_ and 95% air. Cells were routinely tested for the absence of mycoplasma using a MycoAlertTM PLUS Mycoplasma Detection Kit (Lonza, Basel, Swiss). Primary murine pre-B cells were maintained in Opti-MEM medium (Invitrogen) supplemented with 10% FBS, 2 mM L-glutamine, 100 U/mL penicillin, 100 μg/mL streptomycin, and 10 ng/ml mouse IL-7 (R&D Systems, Minneapolis, MN). In some experiments, Hif1α inhibitor Echinomycin (2 nM, Abcam, Cambridge, MA, USA) or mTORC2 inhibitor JR-AB2-011 (1 μM, MedChemexpress, Shanghai, China) were added in culture.

In order to test the infection efficiency of lentivirus on pre-B cells, we first used EGFP-labeled lentivirus to infect pre-B cells. Cells (1 × 10^6^/well) were seeded in 96-well plates. The EGFP-labeled lentivirus particles were added at multiplicity of infection (MOI of 50), and cultured for 12 h before medium was changed. After 24 h, we tested the cell infection efficiency by flow cytometry, and found that the infection efficiency of pre-B cells reached more than 98% (Supplementary Fig. S1B). Then, to overexpress miR-582 or its antagonist in B cells, sequences of pre-miR-582 and anti-miR-582 (Supplementary Fig. S1C, D) were inserted into the lentivirus vector GV369 or GV280, respectively. Viral particles were packaged via a commercial service provided by Genechem (Shanghai, China). The more detail methodology that used to produce the lentivirus are refer to the lentivirus packaging system instructions of Genechem (Shanghai, China). Cells (1 × 10^6^/well) were seeded in 96-well plates. The lentivirus particles were added at multiplicity of infection (MOI of 50), and cultured for 12 h before medium was changed and cultured further for 1–5 days according to experimental designs. The anti-miR-582 lentivirus included anti-miR-582-3p and anti-miR-582-5p at a ratio of 1:1. In each experiment setting, before studying the regulatory effects of pre-miR-582 and anti-miR-582 lentivirus on pre-B cells, we will first determine the expression of pre-miR-582 and anti-miR-582 on pre-B cells.

The 3′-UTR fragments of Rictor (8970-9176, NM_030168) or Hif1α (1193-1399, NM_010431) were amplified by RT-PCR, and inserted into GV272 downstream to the luciferase gene to construct GV272-WT-Rictor and GV272-WT-Hif1α, respectively. The putative binding sites for miR-582 were mutated by PCR to obtain GV272-mut-Rictor and GV272-mut-Hif1α. All constructs were confirmed by DNA sequencing. For luciferase reporter assay, cells were co-transfected with 50 nM of pre-miR-582 mimics or pre-miR-Ctrl, 100 ng reporter construct or GV272, and 5 ng phRL-TK using Lipofectamine 2000^TM^ (Invitrogen) following the manufacturer’s protocol. Cells were harvested 72 h after transfection, and firefly and renilla luciferase activities were evaluated with a Dual-Luciferase Reporter Assay System (Promega).

### Cell proliferation and apoptosis

Pre-B cells were resuspended in PBS and incubated with 5,6-carboxyfluorescein diacetate succinimidyl ester (CFSE) at the concentration of 2.5 μM (Molecular Probes, ThermoFisher Scientific, Waltham, MA, USA) for 15 min at 37 °C, followed by adding 10% FBS-containing Opti-MEM medium to quench CFSE. After washing twice with medium, cells were resuspended in culture medium and cultured in 96-well U bottom plates (5 × 10^5^ cells/well, Corning Inc., Corning, NY, USA) for 5 days, and the proliferation was analyzed by flow cytometry.

To evaluate apoptosis, cells were resuspended in 100 μL binding buffer, prior to the addition of 5 μL Annexin V (BD Bioscience) and incubated for 15 min. Cells were washed twice, and 400 μL of binding buffer and 5 μL 7-AAD were added. The percentage of apoptotic cells were analyzed by flow cytometry.

### Glucose uptake assay

Cells were pelleted at 500 g for 5 min at 4 °C and washed twice with glucose-free medium. The 2-deoxy-2-[(7-nitro-2,1,3-benzoxadiazol-4-yl) amino]-d-glucose (2-NBDG, Cayman Technologies) was diluted to 150 μg/mL in glucose-free medium, and 100 μL of the 2-NBDG-containing medium was added to cells and incubated at 37 °C for 60 min in dark. The cells were washed twice with PBS to remove residual 2-NBDG and then analyzed by flow cytometry.

### Quantitative reverse transcription-PCR (qRT-PCR)

Total RNA was isolated using the Trizol reagent (TaKaRa Biotechnology, Dalian, China) according to the manufacturer’s protocol [25]. cDNA synthesis and quantitative PCR were performed using the Mir-X^TM^ miRNA First-Strand Synthesis Kit (TaKaRa), SYBR Prime Script RT-PCR Kit (TaKaRa) and QuantStudio 3 realtime PCR system (Life Technologies, Waltham, MA), with β-actin and U6 as reference controls for mRNA and miRNA, respectively. Primers are listed in Supplementary Table S1.

### Western blotting

Cells were harvested and washed twice with cold PBS, and lysed using the radio immunoprecipitation assay (RIPA) buffer (Beyotime, Shanghai, China) containing 10 nM phenylmethanesulfonyl fluoride (PMSF). Protein concentrations were determined by a BCA protein assay kit (ThemoFisher Scientific). Samples were separated with sodium dodecyl sulfate-12% polyacrylamide gel electrophoresis (SDS-PAGE), and blotted onto a polyvinylidene fluoride (PVDF) membrane (Millature Antiipore, Billerica, MA). Membranes were blocked with 5% skim milk for 1 h, and incubated with primary antibody at 4 °C overnight followed by secondary antibody for 1 h at room temperature. Antibodies included β-actin (1:2000, Beyotime), Rictor (1:1000, Abcam), Hif1α (1:1000, Abcam), Glut1 (1:1000, Abcam), p-AKT (1:1000, Abcam), AKT (1:1000, Abcam), p-FoxO1 (1:1000, CST), FoxO1 (1:1000, CST), HRP-conjugated goat anti-rabbit IgG (Genshare, Xian, China), and HRP-conjugated goat anti-mouse IgG (Genshare, Xian, China).

### Statistical analysis

Statistical analyses were performed with GraphPad Prism 7.0 software. Student’s two-tailed *t*-test and one-way ANOVA were used to calculate statistical significance. All results are expressed as mean ± SEM. *P* < 0.05 was considered statistically significant.

## Results

### miR-582 ablation expands B cell population in BM and spleen

**Fig. 1:**
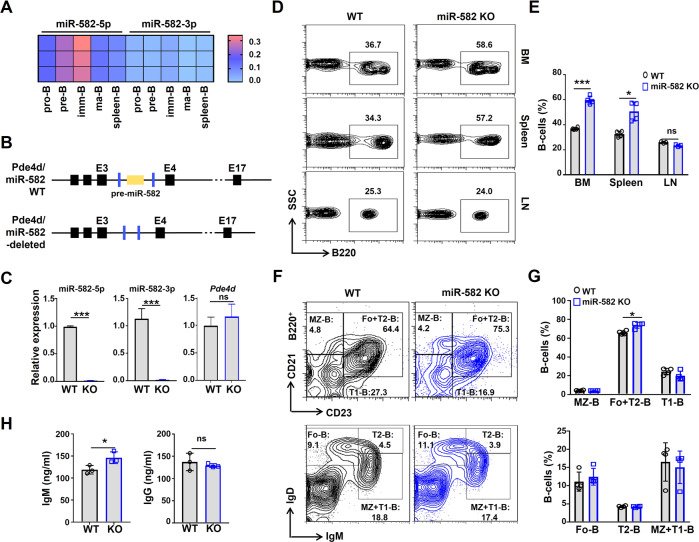
miR-582 ablation leads to B cell expansion in BM and spleen. **A** Expression of miR-582-5p and miR-582-3p in FACS-sorted murine pro-B, pre-B, imm-B, ma-B, and spleen B cells was determined by qRT-PCR. **B** Strategy of CRISPR-Cas9-mediated deletion of the *pre-miR-582* gene, which is accommodated in the third intron of *Pde4d* gene, in mice. Black boxes and lines indicate exons and introns of the murine *Pde4d* gene, respectively. *pre-miR-582* gene is represented with a yellow box, and the blue boxes indicate the position of primers for genotyping. **C** Expression of miR-582-5p, miR-582-3p, and *Pde4d* gene in total BM B cells from wild type and miR-582-deleted mice was determined by qRT-PCR, with U6 and β-actin as reference controls for miRNAs and *Pde4d*, respectively (*n* = 3). **D**, **E** Single cell suspensions from BM, spleen, and lymph nodes (LN) from miR-582 deficient and control mice were analyzed by flow cytometry (**D**), and the percentage of B220^+^ B cells in each organ were quantitatively compared (**E**) (*n* = 4). **F**, **G** Spleen cells from adult miR-582 deficient mice and normal littermates were stained as indicated and analyzed by FACS (**F**). Marginal zone (MZ-) B cells (CD45^+^B220^+^CD21^high^CD23^−^), follicular (Fo-) B cells (CD45^+^IgD^+^IgM^−^), transitional 1 B cells (CD45^+^B220^+^CD21^low/−^CD23^−^), transitional 2 B cells (CD45^+^IgD^high^IgM^+^), Fo+T2-B cells (CD45^+^B220^+^CD21^+^CD23^+^), and MZ + T1-B cells (CD45^+^IgD^low/−^IgM^+^) were quantitatively compared (**G**) (*n* = 4). **H** Serum IgM and IgG in miR-582 KO and control mice were analyzed by ELISA (*n* = 3). Bars represent means ± SEM, *P < 0.05, ***P < 0.001, ns *P* > 0.05.

miR-582 was originally identified as a miRNA expressed in the mouse central nervous system in our lab and likely involved in regulating early neural development (data not shown). To evaluate whether miR-582 regulates B cell development, we examined the expression of miR-582 and its host gene phosphodiesterase (Pde) 4d in B cells at different developmental stages. To do this, pro-B, pre-B, immature (imm)-B, and mature (ma)-B cells were sorted from BM and spleen, and the expression of miR-582-5p, miR-582-3p, and *Pde4d* mRNA was determined by qRT-PCR. The results showed that while miR-582-3p could be detected at low level, miR-582-5p is expressed dynamically along B cell development, with the highest expression detected in pre-B and imm-B cells (Fig. 1A). The mRNA expression of *Pde4d* is inconsistent with miR-582, suggesting that they are regulated by distinct mechanisms (Supplementary Fig. S2A).

To define functions of miR-582 in vivo, we generated miR-582 knockout (KO) mice using the CRISPR/Cas9 technology (Fig. 1B and Supplementary Fig. S2B). QRT-PCR showed that in BM of homozygous miR-582 KO mice, the expression of miR-582-5p and miR-582-3p was almost undetectable, while the expression of *Pde4d* mRNA was not influenced (Fig. 1C). We then analyzed the major hematopoietic populations in BM, spleen and lymph nodes (LN) by FACS. The results showed that B220^+^ B cell population expanded moderately but significantly in BM and spleen, but not LN, of miR-582 KO mice (Fig. 1D, E). Other hematopoietic populations, including T cells (CD3^+^), natural killer (NK) cells (NK1.1^+^), and myeloid cells (CD11b^+^), showed no significant difference in BM or LN between miR-582 KO and WT control mice. Still, we can see a tendency of reductions in spleen T, myeloid, and NK lineages, although not statistically significant (Supplementary Fig. S3A–F). In BM, the relative expression of miR-582-5p in B cells is significantly higher than in T cells and NK cells (Supplementary Fig. S3G).

We then examined B cell subpopulations in the spleen of miR-582 KO and control mice. The result showed that miR-582 ablation resulted in a subtle increase in the percentage of follicular (Fo) and transitional (T) 2 B cells in the spleen (Fig. 1F, G). Serum IgM increased slightly but significantly, while IgG kept unchanged in miR-582 KO mice as compared with the WT control (Fig. 1H). These results suggested that miR-582 is involved primarily in early B cell, most likely pre-B to imm-B, development.

### miR-582 disruption increases BM B cells in a cell-autonomous manner

**Fig. 2:**
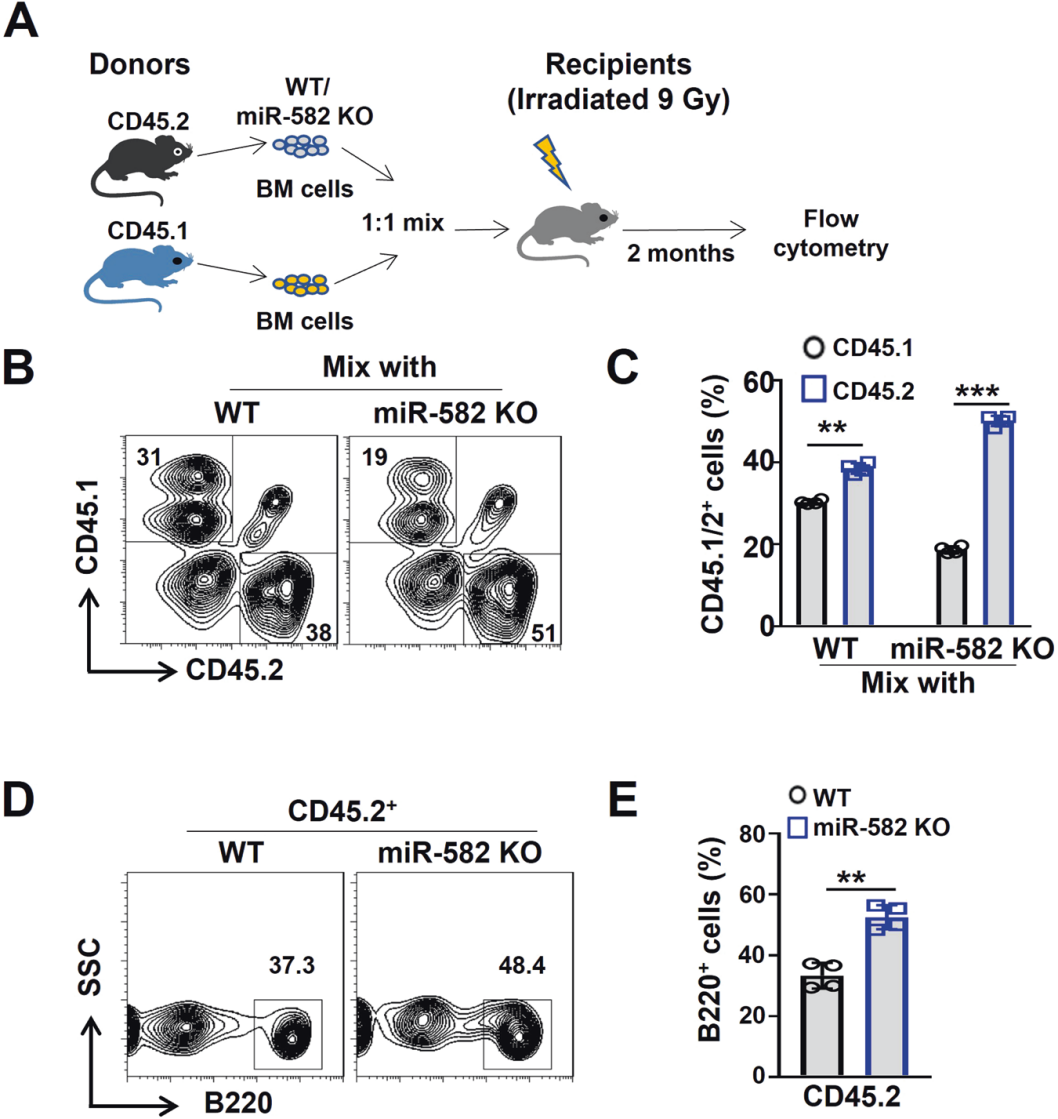
BM B cells expands cell-autonomously in miR-582 deficient mice. **A** Scheme of the competitive BM transplantation experiment. **B**, **C** BM cells from the recipient mice were analyzed for CD45.1^+^ and CD45.2^+^ populations by flow cytometry (*n* = 4). **D**, **E** BM cells from the recipient mice were analyzed for B220^+^ B cells in the CD45.2^+^ populations by flow cytometry and quantitatively compared (*n* = 4). Bars represent means ± SEM, **P < 0.01, ***P < 0.001.

To clarify whether miR-582 disruption increased BM B cells by influencing B cell-intrinsic mechanisms or by modifying BM microenvironment, we performed a competitive BM transplantation assay (Fig. 2A). BM nucleated cells were collected from miR-582 KO or WT control mice (CD45.2), mixed with BM cells from SJL mice (CD45.1) at a ratio of 1:1, and transplanted into lethally irradiated (9 Gy) mice. BM from the recipient mice were analyzed by flow cytometry at 8 weeks of post-transplantation. The result showed that compared with WT mice, CD45.2^+^ BM cells from miR-582 KO mice exhibited a significantly increased proportion over the CD45.1^+^ BM cells (Fig. 2B, C). Moreover, in the CD45.2^+^ population, proportion of B220^+^ B cells from miR-582 KO mice was significantly higher than that from WT mice (Fig. 2D, E). In various hematopoietic cells of bone marrow, miR-582 is relatively highly expressed in B cells, and miR-58 ablation results in increased BM B cells via B cell-intrinsic but not environmental cues.

### miR-582 ablation increases pre-B cells in BM

**Fig. 3:**
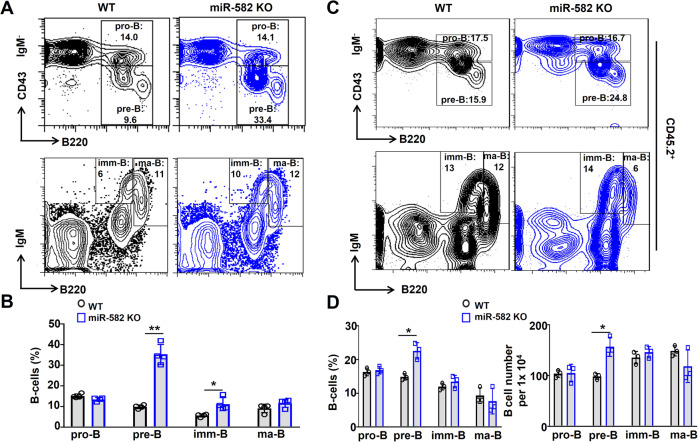
miR-582 deficiency specifically increases pre-B cells in BM. **A**, **B** BM cells from miR-582 KO and control mice were analyzed by FACS for pro-B cells, pre-B cells, imm-B cells, and ma-B cells. B cells in each population were compared (*n* = 4). **C**, **D** Competitive BM transplantation was performed as in Fig. 2A. BM cells were analyzed by FACS for CD45.2^+^ pro-B, pre-B cells, imm-B, and ma-B cells 2 months after the transplantation. CD45.2^+^ B cells in each population and cell numbers were compared (*n* = 3–4). Bars represent means ± SEM, *P < 0.05, **P < 0.01.

In BM, B cells develop through a defined lineage composed of pro-B cells (CD45^+^B220^+^CD43^+^IgM^−^), pre-B cells (CD45^+^IgM^−^B220^+^CD43^−^), imm-B cells (CD45^+^B220^low^IgM^+^) and ma-B cells (CD45^+^B220^high^IgM^+^) [24]. To reveal which developmental stage was influenced under miR-582 deficiency, we analyzed proportion of each B cell population in BM of miR-582 KO and WT mice. The gating strategy for bone marrow B cell subsets is shown in Supplementary Fig. S4. The result showed that pre-B and imm-B cells were significantly increased in miR-582 KO mice as compared with the WT control, while there were no significant changes in BM pro-B and ma-B cells between miR-582 KO and WT control mice (Fig. 3A, B). A similar pattern of B cell subsets was observed in recipient mice of BM transplantation experiment, except imm-B cells population and numbers (Fig. 3C, D). These results indicate that miR-582 deficiency specifically increases pre-B cells in BM.

### miR-582 represses proliferation and promotes apoptosis of pre-B cells

**Fig. 4:**
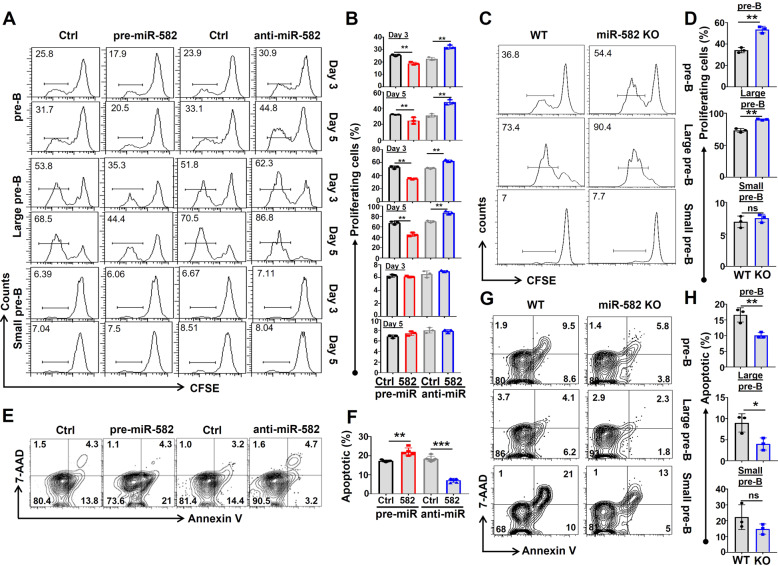
miR-582 overexpression inhibits proliferation and promotes survival of pre-B cells in vitro. **A**, **B** Total pre-B, large pre-B and small pre-B cells were infected with pre-miR-582 or anti-miR-582 lentivirus or their controls, and cultured for 5 days. Cell proliferation was determined using the CFSE labeling assay (*n* = 3). **C**, **D** Total pre-B, large pre-B, and small pre-B cells were harvested from WT and miR-582 KO mice and cultured for 72 h in vitro. Cell proliferation was determined using the CFSE labeling assay (*n* = 3). **E**, **F** pre-B cells were infected with pre-miR-582 or anti-miR-582 lentivirus or their controls, and cultured for 72 h. Apoptosis was evaluated by FACS after staining for Annexin V and 7-AAD. The percentages of apoptotic (Annexin V^+^/7-AAD^−/+^) pre-B cells were quantitatively compared (*n* = 5–6). **G**, **H** Total pre-B, large pre-B, and small pre-B cells were harvested from WT and miR-582 KO mice and cultured for 72 h in vitro. Apoptotic cells were determined by flow cytometry after Annexin V and 7-AAD staining and quantitatively compared (*n* = 3). Bars represent means ± SEM, *P < 0.05, **P < 0.01, ***P < 0.001, ns *P* > 0.05.

At the pre-B stage, B cells undergo vigorous proliferation to expand cell population before entering negative selection through apoptosis, clonal anergy, or BCR editing [26]. The enlarged pre-B population in miR-582 KO mice suggests that miR-582 represses pre-B cell proliferation. To verify this assumption, pre-B cells from wild type mice transfected with pre-miR-582 or miR-582 siRNA (anti-miR-582) using lentivirus, the qRT-PCR results showed that, compared with control group, miR-582 is significantly increased in pre-miR-582 group and decreased in anti-miR-582 group (Supplementary Fig. S6A). Then, cell proliferation and apoptosis were accessed. CFSE labeling assay showed that on day 3 and day 5 after viral infection, the proliferation of pre-B cells, large pre-B cells decreased significantly in the miR-582-overexpressed group (Fig. 4A, B). In contrast, transfection of anti-miR-582 with lentivirus resulted in increased pre-B cell, large pre-B cells proliferation (Fig. 4A, B). Furthermore, we cultured BM B cells from WT and miR-582 KO mice in vitro for 72 h, and analyzed the proliferation of pre-B cells. CFSE labeling assay showed that the proliferation of pre-B, large pre-B cells increased significantly in the miR-582 KO group (Fig. 4C, D). The gating strategy of pre-B cells from enriched bone marrow B cells is showed in Supplementary Fig. S5. We also addressed pre-B cell apoptosis under the same experimental settings by staining cells with Annexin V and 7-AAD. The result showed that compared with the control, there was a significant increase in the proportion of early and late apoptotic (Annexin V^+^/7-AAD^−/+^) pre-B cells upon transfection with pre-miR-582, and pre-B cell apoptosis decreased significantly in the anti-miR-582-transfected group (Fig. 4E, F). Furthermore, we cultured BM B cells from WT and miR-582 KO mice in vitro for 72 h, and analyzed apoptosis by staining with Annexin V and 7-AAD in total pre-B, large pre-B, and small pre-B cell compartments. The results showed that the majority of the decreased apoptotic pre-B cells are large pre-B cells in the miR-582 KO group (Fig. 4G, H). Collectively, these results indicate that miR-582 represses proliferation and promotes apoptosis of pre-B cells, especially the large pre-B cells.

### miR-582 downregulates Rictor and Hif1α by targeting their 3′UTR in pre-B cells

**Fig. 5:**
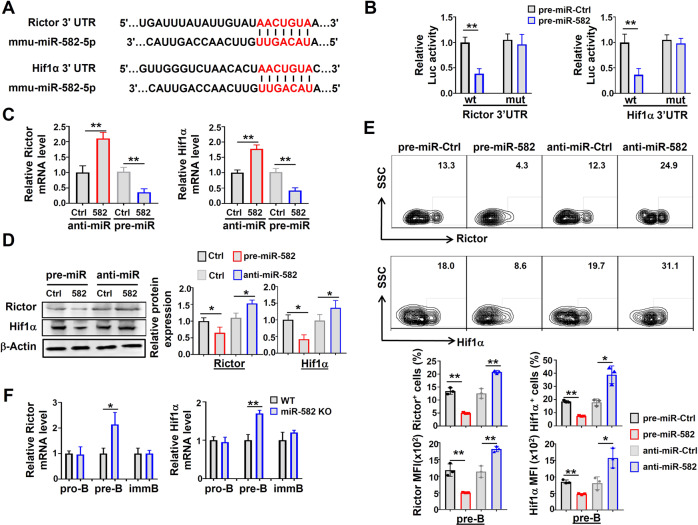
miR-582 downregulates Rictor and Hif1α by directly targets their 3′UTR in pre-B cells. **A** Aligment of the seed sequence of murine miR-582-5p with murine Rictor and Hif1α 3′UTR. Complementary bases are marked with red color. **B** HEK293T cells were transfected with miR-582-5p and different 3′UTR reporters of Rictor or Hif1α for 72 h. Luciferase activity in cell lysates were determined by the dual luciferase reporter assay (*n* = 5). **C**, **D** pre-B cells were infection with pre-miR-582 or anti-miR-582 lentivirus or their controls for 72 h, and Rictor and Hif1α mRNA and protein were determined by qRT-PCR (**C**) and Western blotting (**D**), respectively, with β-actin as an internal control (*n* = 5–6). **E** Pre-B cells were infected with pre-miR-582 or anti-miR-582 lentivirus or their controls. The expression of Rictor and Hif1α in pre-B cells was determined by FACS after cytoplasmic staining, and quantitatively compared using the median fluorescence intensity (MFI) and the proportion of positive cells (*n* = 3). **F** Pro-B, pre-B, and imm-B cells were FACS-purified from the control and miR-582 KO mice, and the expressions of Rictor and Hif1α was determined by qRT-PCR (*n* = 3). Bars represent means ± SEM, *P < 0.05, **P < 0.01.

To address the molecular mechanism by which miR-582 regulates pre-B cell proliferation and apoptosis, we analyzed putative target genes of miR-582 in the TargetScan 6.2 and miRanda database. The results showed that the 3′UTRs of Rictor and Hif1α harbor highly conserved miR-582 binding sites (Fig. 5A). To confirm that miR-582 inhibits Rictor and Hif1α expression, we constructed reporter constructs using wild type and miR-582-binding site-mutated 3′UTR of Rictor and Hif1α (Supplementary Fig. S6B). HEK293T cells, pre-B cells, pro-B cells, and imm-B cells were co-transfected with pre-miR-582 and different reporter plasmids, and luciferase activity was determined 72 h after. The results showed that miR-582 overexpression significantly repressed the luciferase activity derived from reporters of wild type but not mutated Rictor and Hif1α 3′UTRs in HEK293T cells and pre-B cells (Fig. 5B and Supplementary Fig. S6C, D). These results suggest that Rictor and Hif1α are targets of miR-582 in pre-B cells, and the direct regulation of miR-582 on Rictor and Hif1α is a tissue-specific manner. During the early development of B cells, miR-582 may be specifically participate in the regulation of pre-B cell proliferation, providing sufficient cells for later development.

To further determine the effects of miR-582 on the expression of Rictor and Hif1α, pre-B cells were infected with pre-miR-582 or anti-miR-582 lentivirus, and the expression of Rictor and Hif1α was determined. The results showed that overexpression of miR-582 significantly inhibited the mRNA expression of Rictor and Hif1α, while inhibition of miR-582 by anti-miR-582 increased their mRNA levels (Fig. 5C). Western blotting and flow cytometry confirmed that overexpression of miR-582 significantly inhibited the protein expression of Rictor and Hif1α, while anti-miR-582 increased Rictor and Hif1α protein expressions in pre-B cells in vitro (Fig. 5D, E). We then examined the expression of Rictor and Hif1α in B cells of different developmental stages from WT and miR-582 KO mice. The result showed that the mRNA expression of Rictor and Hif1α increased significantly in pre-B cells from miR-582 KO mice (Fig. 5F). Taken together, these results verified that miR-582 directly targets the 3′UTR of Rictor and Hif1α to inhibit their expressions in pre-B cells.

### miR-582 attenuates pre-B cell proliferation and survival by inhibiting Hif1α/Glut1 and mTORC2/AKT signaling pathways

**Fig. 6:**
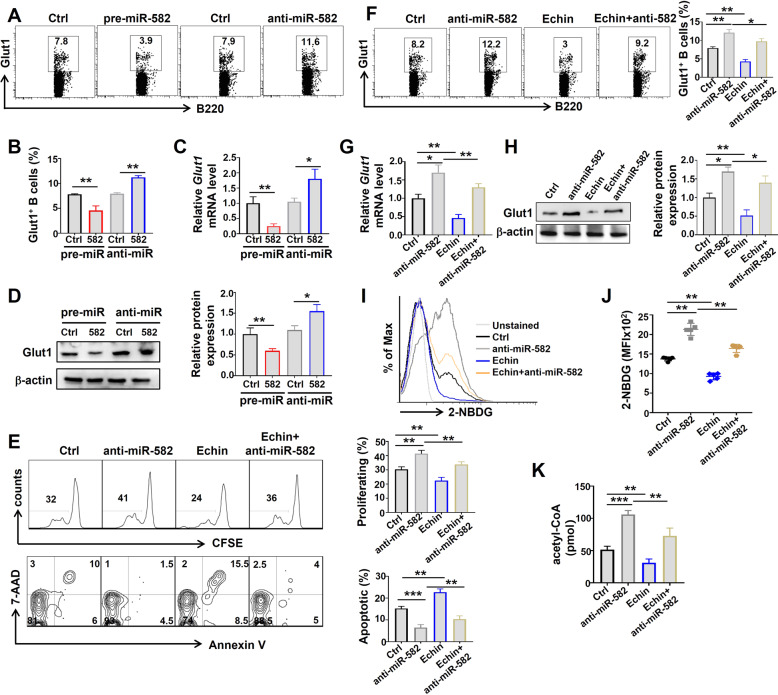
miR-582-mediated modulation of pre-B cell proliferation and survival is dependent on Hif1α regulating Glut1. **A**–**D** miR-582 regulates Hif1α downstream gene, *Glut1*. Pre-B cells were infected with pre-miR-582 or anti-miR-582 lentivirus or their controls for 72 h, and Glut1 expression was determined by flow cytometry (**A**, **B**), qRT-PCR (**C**), and Western blotting (**D**) (*n* = 3–6). **E** Pre-B cells were infected with anti-miR-582 lentivirus and cultured in the presence of the Hif1α inhibitor Echinomycin (Echin) for 72 h. Cell proliferation and apoptosis were evaluated by CFSE labeling and Annexin V^+^ and 7-AAD^+^, respectively, followed by flow cytometry, and quantitatively compared (*n* = 4). **F**–**H** Pre-B cells were infected with anti-miR-582 lentivirus and cultured in the presence of Echin for 72 h. Glut1 expression was determined by flow cytometry (**F**), qRT-PCR (**G**), and Western blotting (**H**) (*n* = 4–6). **I**, **J** Pre-B cells were infected with anti-miR-582 lentivirus and cultured in the presence of Echin for 72 h. 2-NBDG was added to glucose-free medium for 1 h and Intracellular 2-NBDG was analyzed by flow cytometry and quantitatively compared using MFI (*n* = 5). **K** Pre-B cells were infected with anti-miR-582 lentivirus and cultured in the presence of Echin for 72 h. Acetyl-CoA in cell lysates was evaluated and compared (*n* = 6). Bars represent means ± SEM, *P < 0.05,**P < 0.01, ***P < 0.001.

Previous reports have shown that Hif1α and mTORC2 promote pre-B cell proliferation and survival by regulating metabolism [13, 27]. To further explore the mechanisms underlying miR-582 to regulate pre-B cell proliferation and survival, we first examined the effects of miR-582 overexpression or knockdown on the expression of Glut1, a Hif1α downstream molecule. Thus, pre-B cells were infected with pre-miR-582 or anti-miR-582 lentivirus for 72 h, and the expression of Glut1 was examined at different levels. The results showed that overexpression of miR-582 inhibited the mRNA and protein expression of Glut1, while interference of miR-582 expression upregulated the mRNA and protein expression of Glut1 (Fig. 6A–D). We then transfected pre-B cells with anti-miR-582, and cultured cells in the presence of the Hif1α inhibitor, Echinomycin. The result showed that Echinomycin abrogated the effects of miR-582 interference on promoting pre-B cell proliferation and survival (Fig. 6E). Consistently, anti-miR-582-upregulated Glut1 expression was reversed by Echinomycin at mRNA and protein levels (Fig. 6F–H), and glucose uptake and acetyl-CoA content, which were increased upon anti-miR-582 transfection, also decreased (Fig. 6I–K). These results suggest that Hif1α is required in miR-582 ablation-mediated pre-B cell proliferation and survival.

**Fig. 7:**
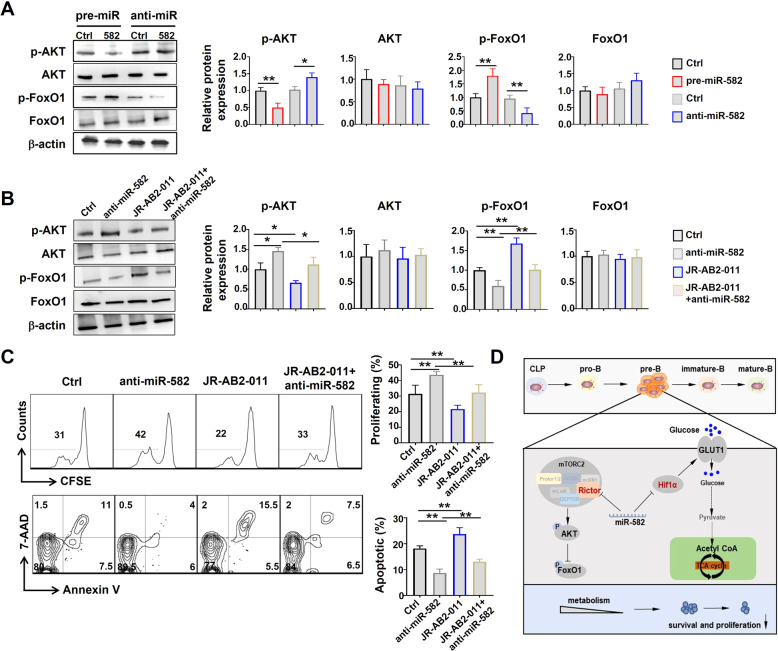
miR-582 simultaneously targets mTORC2/AKT signaling to attenuate pre-B cell proliferation and survival. **A** Pre-B cells were infected with anti-miR-582 lentivirus and cultured for 72 h. The level of p-AKT, AKT, p-FoxO1, and FoxO1 was determined by Western blotting with β-actin as a reference control (*n* = 4–5). **B** Pre-B cells were infected with anti-miR-582 lentivirus and cultured in the presence of the mTORC2 inhibitor JR-AB2-011 for 72 h. The level of p-AKT, AKT, p-FoxO1, and FoxO1 was determined by Western blotting with β-actin as a reference control (*n* = 4). **C** Pre-B cells were infected with anti-miR-582 lentivirus and cultured in the presence of the mTORC2 inhibitor JR-AB2-011 for 72 h. Cell proliferation and apoptosis were evaluated by CFSE labeling and Annexin V^+^ and 7-AAD^+^, respectively, followed by flow cytometry, and quantitatively compared (*n* = 3). **D** The signaling pathway of miR-582 regulate the proliferation and survival of pre-B cell. Bars represent means ± SEM, *P < 0.05, **P < 0.01.

Similarly, infection of pre-B cells with pre-miR-582 lentivirus inhibited phosphorylation (p-AKT) while promoted FoxO1 phosphorylation (p-FoxO1); and interfering miR-582 with anti-miR-582 lentivirus exhibited opposite effects (Fig. 7A). A mTORC2 inhibitor, JR-AB2-011, reversed the effects of anti-miR-582 on AKT and FoxO1 phosphorylation (Fig. 7B), and abrogated increased pre-B cell proliferation and survival upon anti-miR-582 lentivirus infection (Fig. 7C). Altogether, these results suggest that miR-582 ablation leads to increased pre-B cell proliferation and survival via Hif1α/Glut1 signaling and mTORC2/AKT signaling pathways (Fig. 7D).

## Discussion

In this study, we report that miR-582 plays an important role in early B cell development. We have found that miR-582 is essential for inhibiting excessive proliferation of pre-B cells during B cell development in BM. We have also identified that the Hif1α/Glut1 signaling and mTORC2/AKT signaling, two metabolism-related signaling pathways, are the downstream targets of miR-582 involved in regulating proliferation and survival of pre-B cells. These findings provide a new regulatory element to harness normal B cell development in BM.

miRNAs regulates target gene expression by inhibiting mRNA translation and/or decay [28]. Previous studies have shown that miR-582 is involved in various biological processes. miR-582-5p affect RUNX3 expression via competitively binding with SNHG5 and regulates osteogenic differentiation and apoptosis of BM mesenchymal stem cells (MSCs) [29]. Recently, miR-582 has also been found to participate in regulating several metabolism-related genes, such as AKT3 and FOXC1, in different biological process [30, 31]. The role and regulation mechanism of miR-582 in tumor development has been well-studied [32, 33]. miR-582-5p serves as an anti-oncogenic biomarker in intermediate risk acute myeloid leukemia with normal cytogenetics and can inhibit proliferation and induce apoptosis of leukemia cells [34]. Besides leukemia, miR-582 has been shown to inhibit the development of solid tumors, including non-small cell lung cancer [35], bladder cancer [36], and human colorectal carcinoma [37], by targeting different gene, such as Hippo-YAP, TTK, and Rab27a. These studies have shown that miR-582 plays a negative regulatory role in tumor progression. Consistently, our study also reveals a negative regulatory role of miR-582 on pre-B cell proliferation. These findings suggest that miR-582 overexpression in tumor cells could be a potential strategy for repressing tumor growth, but an inhibiting effect on the development of early B cells should be taken into account.

Pre-B cells are characterized by extensive cell proliferation, in which metabolism plays an important role [10]. Previous studies have shown that the glycolytic pathway in Hif1α-deficient B220^+^ bone marrow B cells is much less functionally effective than in wild-type control B cells and the development of pre-B cells would been inhibited [13], indicating that Hif1α plays a key role in the glycolysis pathway of BM B cell precursors. In our study, we have provided functional evidence that miR-582 negatively regulates the Hif1α/Glut1 signaling and subsequently results in acetyl-CoA production. Our study also suggests that miR-582 downregulates Hif1α/Glut1 signaling at least via targeting Hif1α and functions as a novel negative regulator of pre-B cell glucose metabolism, that helps to fine-tune the early B cell development. Moreover, previous researches have shown that when mTOR binds with Rictor to form mTORC2, it phosphorylates specific substrates including AKT, that promotes cell survival and proliferation [38]. It is well known that activation of mTORC2 signaling results in phosphorylation and deactivation of FOXO by activating AKT during regulation of cell proliferation and survival [39]. In our study, we have provided functional evidence that miR-582 negatively regulates the mTORC2/AKT signaling pathway and subsequently results in more p-FoxO1 in pre-B cells. Combining previous researches and our results, it is clear that miR-582 downregulates mTORC2/AKT signaling at least via targeting Rictor and functions as a novel negative regulator of pre-B cell proliferation.

In conclusion, our study reported here has uncovered a previously unknown role of miR-582 as a negative regulator of pre-B cell proliferation and survival. Our findings provide novel insights into how miR-582 inhibits the proliferation and survival of pre-B cells by directly targeting Rictor and Hif1α, which likely further regulate metabolism to control pre-B cell expansion during early B cell development. These studies are of significance in understanding not only the regulation of early B cell development, but also pre-B cell-associated human malignancies.

## Supplementary information

supplementary materials

AJ-checklist

## Data Availability

All data needed to evaluate the conclusions in the paper are present in the paper. Additional data related to this paper may be requested from the corresponding author on reasonable request.

## References

[1] Cooper MD (2015). The early history of B cells. Nat Rev Immunol.

[2] Nagasawa T (2006). Microenvironmental niches in the bone marrow required for B-cell development. Nat Rev Immunol.

[3] Seda V, Mraz M (2015). B‐cell receptor signalling and its crosstalk with other pathways in normal and malignant cells. Eur J Haematol.

[4] Winkler TH, Mårtensson IL (2018). The role of the pre-b cell receptor in b cell development, repertoire selection, and tolerance. Front Immunol.

[5] Yam-Puc JC, Zhang L, Zhang Y, Toellner KM (2018). Role of B-cell receptors for B-cell development and antigen-induced differentiation. F1000Research.

[6] Melchers F (2015). Checkpoints that control B cell development. J Clin Investig.

[7] Aurrand-Lions M, Mancini SJ (2018). Murine bone marrow niches from hematopoietic stem cells to B cells. Int J Mol Sci.

[8] Herzog S, Reth M, Jumaa H (2009). Regulation of B-cell proliferation and differentiation by pre-B-cell receptor signalling. Nat Rev Immunol.

[9] Clark MR, Mandal M, Ochiai K, Singh H (2014). Orchestrating B cell lymphopoiesis through interplay of IL-7 receptor and pre-B cell receptor signalling. Nat Rev Immunol.

[10] Stein M, Dütting S, Mougiakakos D, Bösl M, Fritsch K, Reimer D (2017). A defined metabolic state in pre B cells governs B-cell development and is counterbalanced by Swiprosin-2/EFhd1. Cell Death Differ.

[11] Urbanczyk S, Stein M, Schuh W, Jäck HM, Mougiakakos D, Mielenz D (2018). Regulation of energy metabolism during early B lymphocyte development. Int J Mol Sci.

[12] Jellusova J (2020). The role of metabolic checkpoint regulators in B cell survival and transformation. Immunol Rev.

[13] Kojima H, Kobayashi A, Sakurai D, Kanno Y, Hase H, Takahashi R (2010). Differentiation stage-specific requirement in hypoxia-inducible factor-1α-regulated glycolytic pathway during murine B cell development in bone marrow. J Immunol.

[14] Fox CJ, Hammerman PS, Thompson CB (2005). Fuel feeds function: energy metabolism and the T-cell response. Nat Rev Immunol.

[15] Lee K, Heffington L, Jellusova J, Nam KT, Raybuck A, Cho SH (2013). Requirement for Rictor in homeostasis and function of mature B lymphoid cells. Blood J Am Soc Hematol.

[16] Limon JJ, So L, Jellbauer S, Chiu H, Corado J, Sykes SM (2014). mTOR kinase inhibitors promote antibody class switching via mTORC2 inhibition. Proc Natl Acad Sci.

[17] Wang H, Zhang Y, Wu X, Wang Y, Cui H, Li X (2018). Regulation of human natural killer cell IFN-γ production by microRNA-146a via targeting the NF-κB signaling pathway. Front Immunol.

[18] Li J, Wan Y, Ji Q, Fang Y, Wu Y (2013). The role of microRNAs in B-cell development and function. Cell Mol Immunol.

[19] de Oliveira JC, Brassesco MS, Scrideli CA, Tone LG, Narendran A (2012). MicroRNA expression and activity in pediatric acute lymphoblastic leukemia (ALL). Pediatr Blood Cancer.

[20] Ma X, Zhou J, Zhong Y, Jiang L, Mu P, Li Y (2014). Expression, regulation and function of microRNAs in multiple sclerosis. Int J Med Sci.

[21] Li J, Qin Y, Zhang H (2018). Identification of key miRNA‑gene pairs in chronic lymphocytic leukemia through integrated analysis of mRNA and miRNA microarray. Oncol Lett.

[22] Venigalla RK, McGuire VA, Clarke R, Patterson‐Kane JC, Najafov A, Toth R (2013). PDK1 regulates VDJ recombination, cell‐cycle exit and survival during B‐cell development. EMBO J.

[23] Han H, Tanigaki K, Yamamoto N, Kuroda K, Yoshimoto M, Nakahata T (2002). Inducible gene knockout of transcription factor recombination signal binding protein‐J reveals its essential role in T versus B lineage decision. Int Immunol.

[24] Allende ML, Tuymetova G, Lee BG, Bonifacino E, Wu YP, Proia RL (2010). S1P1 receptor directs the release of immature B cells from bone marrow into blood. J Exp Med.

[25] Gao XY, Zang J, Zheng MH, Zhang YF, Yue KY, Cao XL (2021). Temozolomide treatment induces HMGB1 to promote the formation of glioma stem cells via the TLR2/NEAT1/Wnt Pathway in Glioblastoma. Front Cell Dev Biol.

[26] Mårtensson IL, Almqvist N, Grimsholm O, Bernardi AI (2010). The pre-B cell receptor checkpoint. FEBS Lett.

[27] Li M, Lazorchak AS, Ouyang X, Zhang H, Liu H, Arojo OA (2019). Sin1/mTORC2 regulate B cell growth and metabolism by activating mTORC1 and Myc. Cell Mol Immunol.

[28] Huntzinger E, Izaurralde E (2011). Gene silencing by microRNAs: contributions of translational repression and mRNA decay. Nat Rev Genet.

[29] Zheng J, Guo H, Qin Y, Liu Z, Ding Z, Zhang L, et al. SNHG5/miR-582-5p/RUNX3 feedback loop regulates osteogenic differentiation and apoptosis of bone marrow mesenchymal stem cells. J Cell Physiol. 2020. 10.1002/jcp.29527. 10.1002/jcp.29527 33111341

[30] Zhang Y, Huang W, Ran Y, Xiong Y, Zhong Z, Fan X (2015). miR-582-5p inhibits proliferation of hepatocellular carcinoma by targeting CDK1 and AKT3. Tumor Biol.

[31] Wang WW, Chen B, Lei CB, Liu GX, Wang YG, Yi C (2017). miR-582-5p inhibits invasion and migration of salivary adenoid cystic carcinoma cells by targeting FOXC1. Jpn J Clin Oncol.

[32] Xie M, Yu T, Jing X, Ma L, Fan Y, Yang F (2020). Exosomal circSHKBP1 promotes gastric cancer progression via regulating the miR-582-3p/HUR/VEGF axis and suppressing HSP90 degradation. Mol Cancer.

[33] Wang L, Zhang M (2018). miR-582-5p is a potential prognostic marker in human non-small cell lung cancer and functions as a tumor suppressor by targeting MAP3K2. Eur Rev Med Pharmacol Sci.

[34] Wang X, Feng Y, Zhang P, Chen H, Bai J, Wang F (2020). miR-582-5p serves as an antioncogenic biomarker in intermediate risk AML with normal cytogenetics and could inhibit proliferation and induce apoptosis of leukemia cells. Cell Biol Int.

[35] Zhu B, Finch-Edmondson M, Lee Y, Wan Y, Sudol M, DasGupta R (2021). miR-582-5p is a tumor suppressor microRNA targeting the hippo-YAP/TAZ signaling pathway in non-small cell lung cancer. Cancers.

[36] Tian Y, Guan Y, Su Y, Luo W, Yang G, Zhang Y (2020). MiR-582-5p inhibits bladder cancer-genesis by suppressing TTK expression. Cancer Manag Res.

[37] Zhang X, Zhang Y, Yang J, Li S, Chen J (2015). Upregulation of miR-582-5p inhibits cell proliferation, cell cycle progression and invasion by targeting Rab27a in human colorectal carcinoma. Cancer Gene Ther.

[38] Iwata TN, Ramírez-Komo JA, Park H, Iritani BM (2017). Control of B lymphocyte development and functions by the mTOR signaling pathways. Cytokine Growth Factor Rev.

[39] Tripathy S, Jump DB (2013). Elovl5 regulates the mTORC2-Akt-FOXO1 pathway by controlling hepatic cis-vaccenic acid synthesis in diet-induced obese mice. J Lipid Res.

