# Supplementary material for: miR-582 negatively regulates pre-B cell proliferation and survival through targeting Hif1α and Rictor
Source: Cell Death Dis. 2022 Feb 3;13(2):107. doi: 10.1038/s41419-022-04560-y (PMC8814019; doi:10.1038/s41419-022-04560-y)
Supplement: Supplementary file 1 — supplementary materials [file 41419_2022_4560_MOESM1_ESM.docx]

**Supplementary Material**

**Supplementary Table S1. PCR Primers Used in the Study.**

| **Name/Target gene** | **Primer sequence** |
| --- | --- |
| Mouse miR-582-5p | F: 5’-ATACAGTTGTTCAACCAGTTAC-3’ |
| Mouse miR-582-3p | F:5’-GGATGACACGCAAATTCGTGAAGC-3’ |
| Mouse miR-582-5p and 3p | R: mRQ 3’ Primer of Mir-X^TM^ miRNA First-Strand Synthesis Kit |
| U6 | U6 Forward and Reverse primers of Mir-X ^TM^ miRNA First-Strand Synthesis Kit |
| Mouse Pde4d | F: 5’-TTTTGCCAGTGCAATACATGATG-3’  R: 5’-CAGAGCGAGTTCCGAGTTTGT-3’ |
| Mouse Rictor | F: 5’-GCTGCGCTATCTCATCCAAGA-3’  R: 5’-GGGTTCTGAAGTGCTAGTTCAC-3’ |
| Mouse Hif1α | F: 5’-TCTCGGCGAAGCAAAGAGTC-3’  R: 5’-AGCCATCTAGGGCTTTCAGATAA-3’ |
| Mouse Glut1 | F: 5’-TCTCGGCTTAGGGCATGGAT-3’  R: 5’-TCTATGACGCCGTGATAGCAG-3’ |
| Mouse β-actin | F: 5’-CATCCGTAAAGACCTCTATGCCAAC-3’  R: 5’-ATGGAGCCACCGATCCACA-3’ |
| WT-F/Mut-F | 5’-TACACGGTAAAATCAGCTACGCGACA-3’ |
| WT-R | 5’-catggcctctggctgagagaaactt-3’ |
| Mut-R | 5’-ccctcctgcttctacctcttgggat-3’ |
| mmu-Hif1α 3’UTR | F: 5’-GATCGCCGTGTAATTCTAGAATTTTAGGAATATAGAGTTG-3’  R: 5’-CCGGCCGCCCCGACTCTAGATTTTATGAGCTAGAAATGTTG  AG-3’ |
| mmu-Rictor 3’UTR | F:5’-GATCGCCGTGTAATTCTAGAACCTTTTTTTTTTTTTTTTTAG-3’  R: 5’-CCGGCCGCCCCGACTCTAGAAAAGTGAGCAAAACCATTTTG  GG-3’ |

**Supplementary Table S2. Antibodies used in this study.**

| Name | Labeling | Cat.No. | Supplier |
| --- | --- | --- | --- |
| Anti-mouse CD45 | Briliant Violet 510 | 103137 | Biolegend |
| Anti-mouse CD45 | [Brilliant Violet 421](https://www.biolegend.com/en-us/search-results/brilliant-violet-421-anti-mouse-cd45-antibody-7253) | 103133 | Biolegend |
| Anti-mouse CD45.2 | PE | 109807 | Biolegend |
| Anti-mouse CD45.1 | APC | 110713 | Biolegend |
| Anti-mouse CD3 | APC | 100236 | Biolegend |
| Anti-mouse NK1.1 | PE | 156503 | Biolegend |
| Anti-mouse/human CD45R/B220 | PerCP/Cyamine5.5 | 103236 | Biolegend |
| Anti-mouse/human CD45R/B220 | APC | 103211 | Biolegend |
| Anti-mouse/human CD45R/B220 | PE | 103207 | Biolegend |
| Anti-mouse/human CD11b | FITC | 101205 | Biolegend |
| Anti-mouse CD43 | FITC | 143203 | Biolegend |
| Anti-mouse CD43 | PE/Cyanine 7 | 143209 | Biolegend |
| Anti-mouse CD43 | APC | 143207 | Biolegend |
| Anti-mouse IgM | PE | 406508 | Biolegend |
| Anti-mouse IgM | APC/Cyanine 7 | 406515 | Biolegend |
| Anti-mouse CD21/CD35 | FITC | 123407 | Biolegend |
| Anti-mouse CD23 | APC | 101619 | Biolegend |
| Anti-mouse IgD | FITC | 405703 | Biolegend |
| 7-AAD | 7-AAD | 420404 | Biolegend |
| Anti-mouse Rictor | PE | NBP1-51645 | NOVUSBIO |
| Anti-human/mouse Hif1α | PE | IC1935P | R&D system |
| Anti-mouse/human Glut1 | Alexa Fluor® 647 | ab195020 | Abcam |
| Anti-β-actin |  | AF5001 | Beyotime |
| Anti-Rictor |  | ab104838 | Abcam |
| Anti-Hif1α |  | ab216842 | Abcam |
| Anti-Glut1 |  | Ab115730 | Abcam |
| Anti-pAkt (Ser473) |  | 4060T | Cell Signaling |
| Anti-Akt |  | 9272S | Cell Signaling |
| Anti-pFoxO1 (Ser256) |  | 9461S | Cell Signaling |
| Anti-FoxO1 |  | 2880S | Cell Signaling |

**
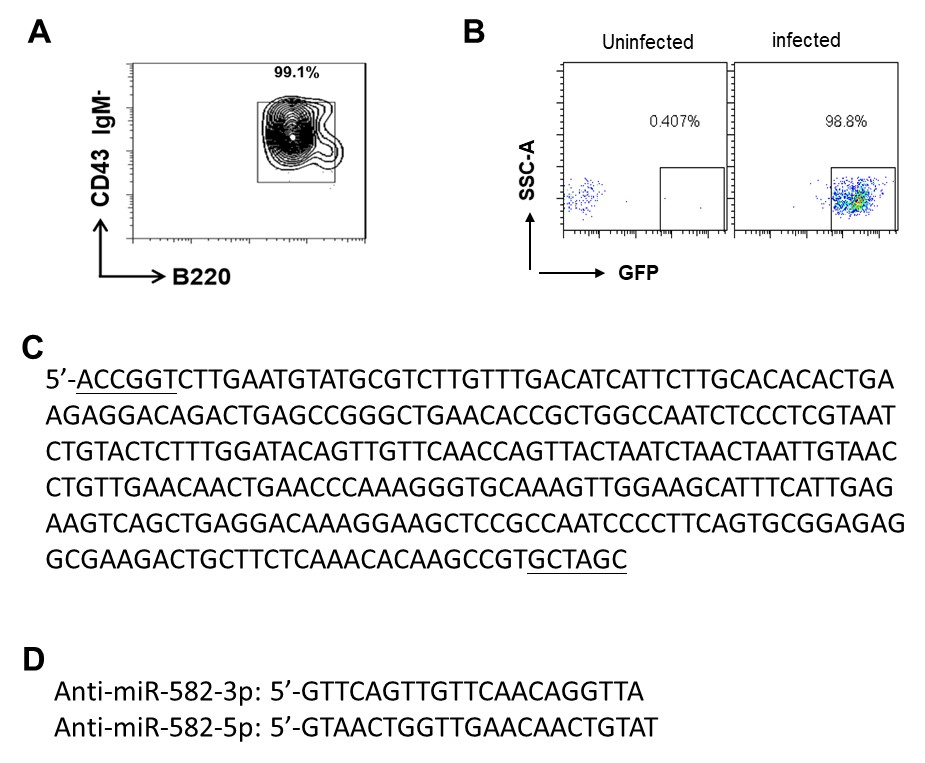
**

**B220**

**CD43**

**IgM^-^**

**Fig. S1.** **(A)** Flow cytometry of sorted pre-B cells. **(B)** Detect the infection efficiency of EGFP-labeled lentivirus on pre-B cells by flow cytometry. **(C, D)** Sequence of pre-miR-582 and anti-miR-582, which were inserted into lentivirus vector for overexpressing and knocking down miR-582, respectively.

**
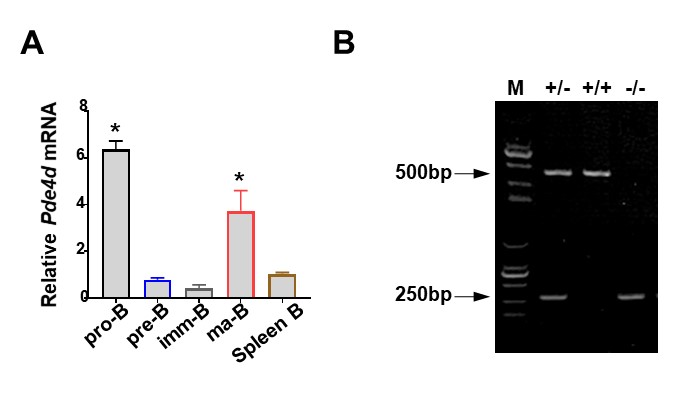
**

**Fig. S2. (A)** Expression of *Pde4d* in pro-B, pre-B, imm-B, ma-B and spleen B cells as determined by qRT-PCR (n = 3-5). **(B)** Genotyping of miR-582 knockout mice. Data are presented as the mean ± SEM, **P <* 0.05.

**
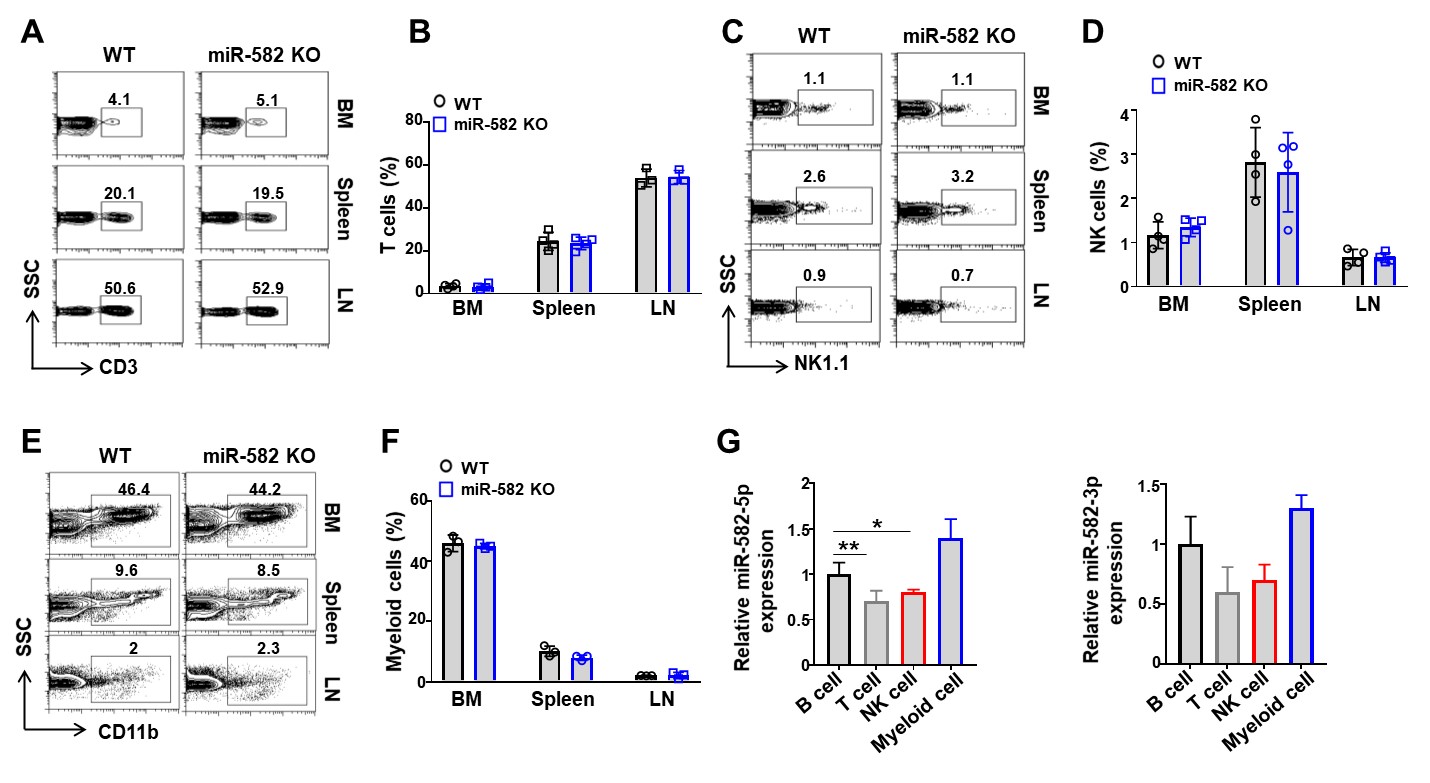
**

**Fig. S3.** miR-582 knockout does not influence T cells, NK cells and myeloid cells in BM and the expression of miR-582-5p in B cells is higher than T cells and NK cells in BM. **(A-F)** BM, spleen and LN cells from miR-582 KO and wild type control mice were analyzed by flow cytometry after staining for T cells (A, B), NK cells (C, D), and myeloid cells (E, F), as indicated. The proportion and number of cells were compared (n = 4). (**G**) The expression of miR-582 in B cells, T cells, NK cells and myeloid cells of BM. Data are presented as the mean ± SEM, **P* < 0.05, ***P* < 0.01.

**
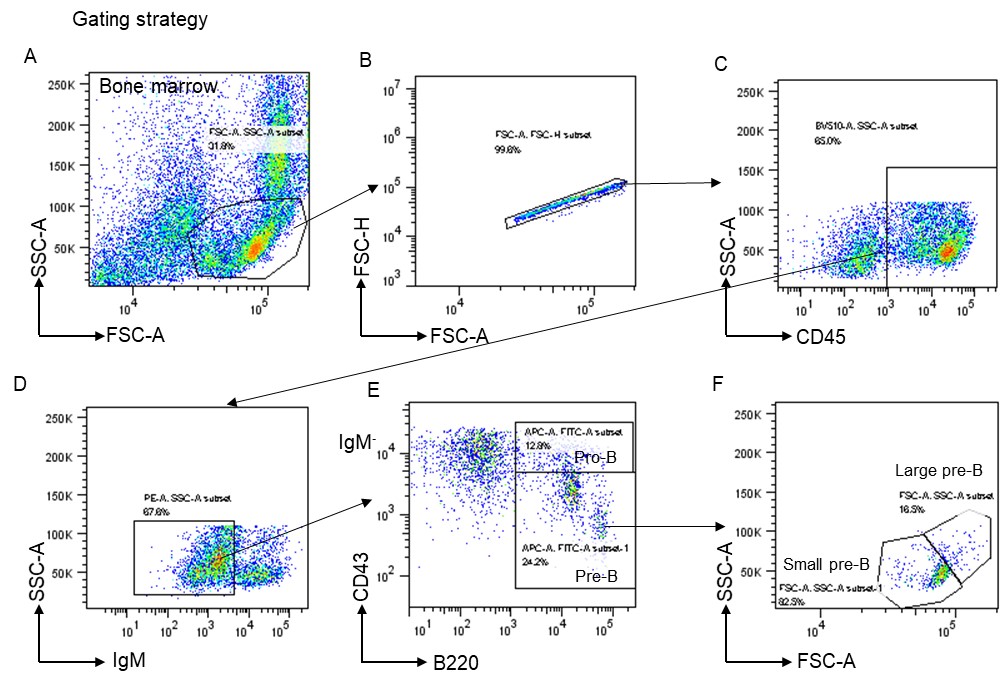
**

**Fig. S4**. The gating strategy of pro-B, pre-B, large pre-B and small pre-B cells.


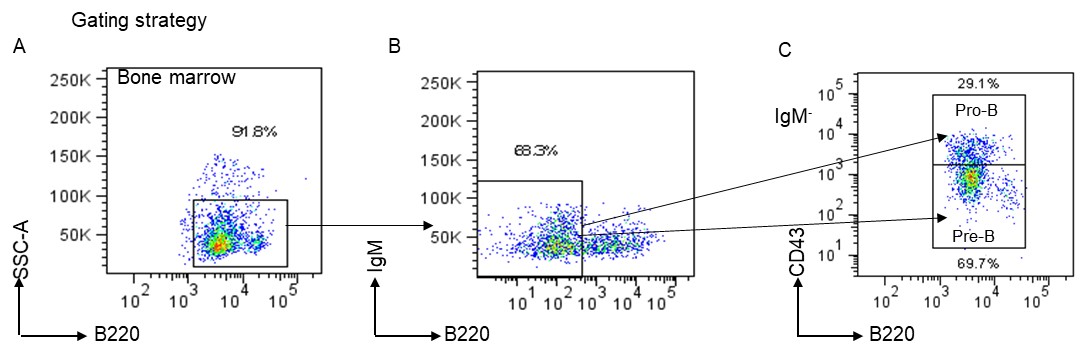


**Fig. S5**. The gating strategy of pro-B, pre-B in enriched bone marrow B cells.

**
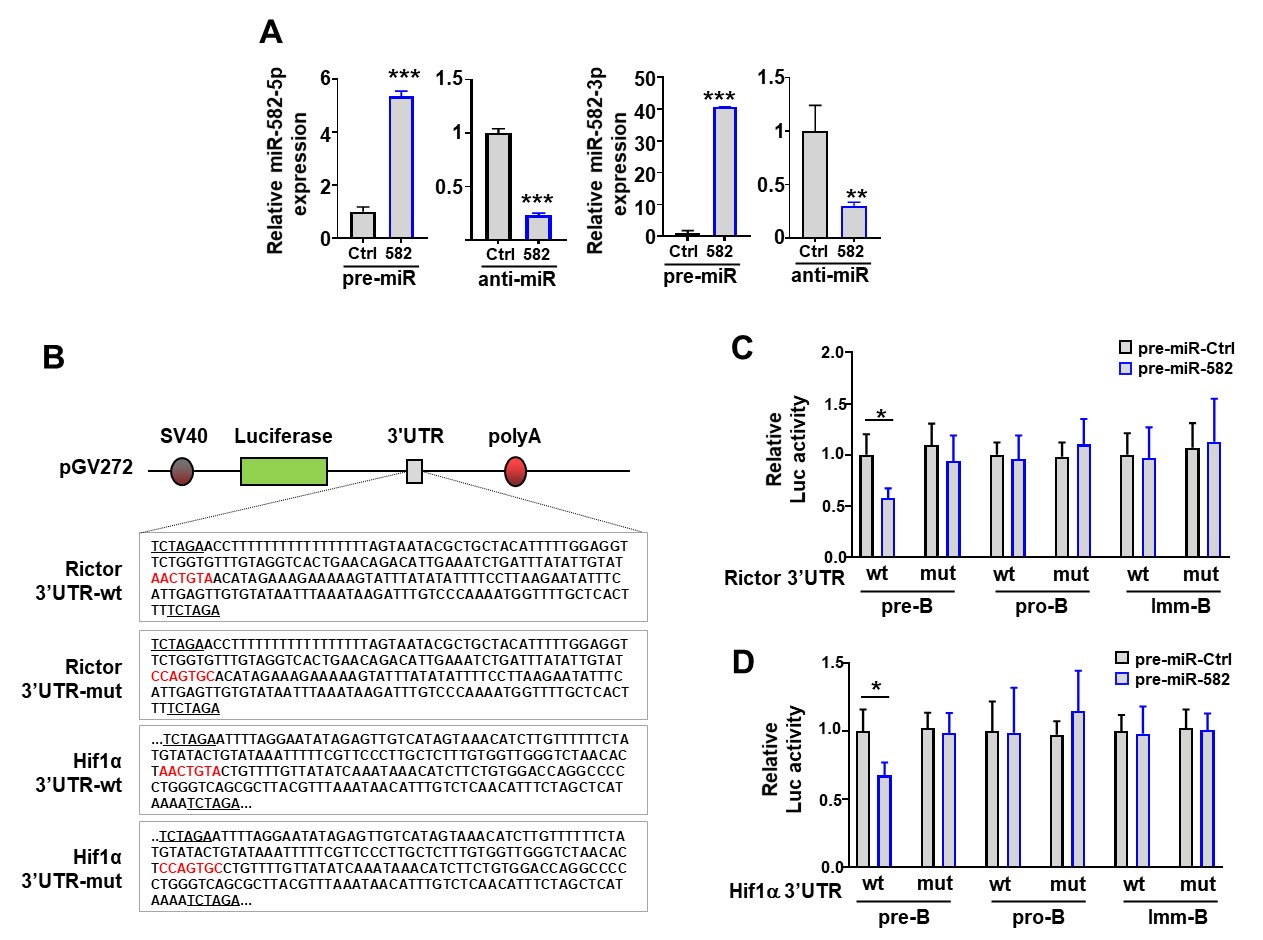
**

**Fig. S6**. Overexpression and knockdown of miR-582. **(A)** pre-B cells were infected with pre-miR-582 or anti-miR-582 lentivirus for 72 h. The level of miR-582 was determined by qRT-PCR. **(B)** The sequences of Rictor and Hif1a 3’UTR used for schematic of the reporter constructs. (C-D) pre-B, pro-B and imm-B cells were transfected with pre-miR-582 and different 3’UTR reporters of Rictor or Hif1α for 72 h. Luciferase activity in cell lysates were determined by the dual luciferase reporter assay (n = 5). Bars represent means ± SEM, **P* < 0.05.
